# MHD flow of Maxwell fluid with nanomaterials due to an exponentially stretching surface

**DOI:** 10.1038/s41598-019-43549-0

**Published:** 2019-05-13

**Authors:** Umer Farooq, Dianchen Lu, Shahzad Munir, Muhammad Ramzan, Muhammad Suleman, Shahid Hussain

**Affiliations:** 1 Department of Mathematics, Faculty of Science, Jiangsu University, 212013 Zhenjiang, China; 2 Department of Mathematics, COMSATS University Islamabad, Park road, Tarlai Kalan, 44000 Islamabad, Pakistan; 3 Department of Mechanical Engineering, Sejong University, Seoul, 143-747 Korea; 4 Department of Computer Science, Bahria University, Islamabad Campus, Islamabad, 44000 Pakistan; 5 School of Materials Science and Engineering, Jiangsu University, Zhenjiang, 212013 China

**Keywords:** Applied mathematics, Software

## Abstract

In many industrial products stretching surfaces and magnetohydrodynamics are being used. The purpose of this article is to analyze magnetohydrodynamics (MHD) non-Newtonian Maxwell fluid with nanomaterials in a surface which is stretching exponentially. Thermophoretic and Brownian motion effects are incorporated using Buongiorno model. The given partial differential system is converted into nonlinear ordinary differential system by employing adequate self-similarity transformations. Locally series solutions are computed using BVPh 2.0 for wide range of governing parameters. It is observed that the flow is expedite for higher Deborah and Hartman numbers. The impact of thermophoresis parameter on the temperature profile is minimal. Mathematically, this study describes the reliability of BVPh 2.0 and physically we may conclude the study of stretching surfaces for non-Newtonian Maxwell fluid in the presence of nanoparticles can be used to obtain desired qualities.

## Introduction

Heat transfer and magnetohydrodynamics in the boundary layer flows are important research topics due to their usage in industry and metallurgy. Several procedures such as chilling of filaments or continuous strips by taking them out from stagnant fluid involve stretching of these strips. The refrigeration of temperature reduction finally defines the quality of the end product. Further such flows are important in view of engineering applications related to geothermal energy extractions, crystal growing, power plants, plasma studies, paper production and MHD generators. Sakiadis^1–3^ presented pioneering research on flow induced by stretching surface. Afterwards, the problems for linear and nonlinear stretching surfaces have been investigated extensively (see for instance)^4–6^. Pioneer research on the flow due to an exponentially stretching surface was done by Magyari and Keller^7^. Sajid and Hayat^8^ discussed the importance of thermal radiation. Sahoo and Poncet^9^ studied partial slip effect for the third grade fluid flow. Mukhopadhyay investigated porous medium and thermally stratified medium in an exponentially stretching surface^10,11^. Rahman *et al*.^12^ addressed such flow considering second order slip by utilizing Buongiorno’s model. Hayat *et al*.^13^ developed analysis for Oldroyd-B fluid. Patil *et al*.^14^ obtained non-similar solutions for such flows for stretching surface considering mixed convection, double diffusion and viscous dissipation effects. Few other studies with reference to the flow and heat transfer characteristics of viscous and non-viscous fluids over surfaces which are stretching exponentially can be found through the refs therein^15–23^.

The studies related to non-Newtonian fluids have generated considerable interest in recent times. This is as a result of their numerous utilizations in industrial products. In general, such fluids cannot be explained by one constitutive equation. Hence, different constitutive equations are proposed in view of the diversity of such fluids. Fluids of non-Newtonian types are mainly distributed among integral, rate and differential types. Maxwell fluid falls under rate type non-viscous fluids category. This class illustrates the relaxation time effects. In past, Fetecau^24^ obtained exact solution for Maxwell fluid flow. Wang and Hayat^25^ studied Maxwell fluid flow in porous medium. Fetecau *et al*.^26^ investigated fraction Maxwell fluid for unsteady flow. Hayat *et al*.^27^ studied two-dimensional MHD Maxwell fluid. Heyhat and Khabzi^28^ investigated MHD upper-convected Maxwell (UCM) fluid flow above a flat rigid region. Hayat and Qasim^29^ obtained series solutions for two-dimensional MHD flow with thermophoresis and Joule heating. Jamil and Fetecau^30^ considered Maxwell fluid for Helical flows between coaxial cylinders. Zheng *et al*.^31^ constructed closed form solutions for generalized Maxwell fluid in a rotating flow. Wang and Tan^32^ presented stability analysis for Maxwell fluid subject to double-diffusive convection, porous medium and soret effects. Motsa *et al*.^33^ examined UCM flow in a porous structure. Mukhopadhyay *et al*.^34^ elaborated thermally radiative Maxwell fluid flow over a continuously permeable expanding surface. Ramesh *et al*.^35^ numerically investigated Maxwell fluid with nanomaterials for MHD flow in a Riga plate. Ijaz *et al*.^36^ explored the behavior of Maxwell nanofluid flow for the motile gyrotactic microorganism in magnetic field. UCM fluid model for non-Fourier heat flux is investigated by Ijaz *et al*.^37^.

Nanofluids consists of ordinary liquids and nanoparticles. Nanofluids are quite useful to improve the performance of ordinary liquids. Nanofluids are developed by inserting fibers and nanometer size particles to original fluids. Nanofluids are especially significant in hybrid powered engines, pharmaceutical processes, fuel cells, microelectronics. The nanoparticles basically connect atomic structures with bulk materials. Commonly used ordinary liquids include toluene, oil, water, engine oil and ethylene glycol mixtures. The metal particles include aluminum, titanium, gold, iron or copper. The nanofluids commonly contains up to 5% fraction volume of nanoparticles to obtain significant improvement in heat transfer. Further, the magnetic nanofluids have great interest in optical switches, biomedicine, cancer therapy, cell separation, optical gratings and magnetic resonance imaging. An extensive review on nanofluids includes the attempts of^38,39^. One can also mention the previous recent studies^40–45^ regarding the improvement of thermal conductivity in the nanofluids.

In view of aforementioned discussion, the MHD flow of non-viscous Maxwell fluid with nanomaterials in an exponentially stretching surface is addressed. The BVPh 2.0 which is developed on the basis of optimal homotopy analysis method (OHAM) is employed to solve nonlinear differential system^46–50^. The convergence of the present results is discussed by the so-called average squared residual errors. Velocity, temperature, concentration, the local Sherwood and the local Nusselt number are also examined through graphs.

## Problem Formulation

**Figure 1:**
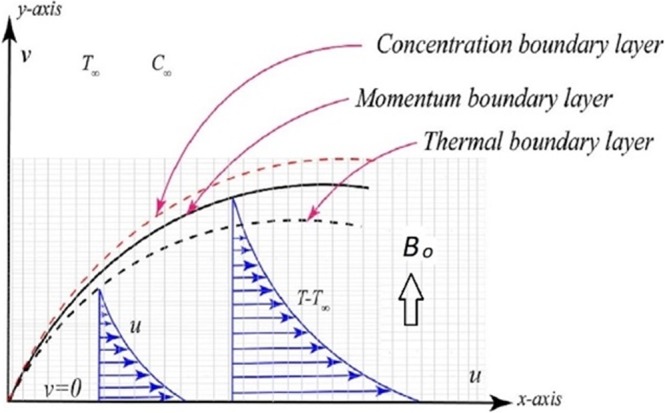
Physical Configuration.

Figure 1 describes the MHD laminar, incompressible flow, thermal and concentration boundary layers in a surface which is stretching exponentially with velocity *U*_*w*_ and given concentration *C*_*w*_ and temperature *T*_*w*_ is described using boundary layer theory as follows:

1 $$\frac{\partial u}{\partial x}+\frac{\partial v}{\partial y}=0,$$

2 $$u\frac{\partial u}{\partial x}+v\frac{\partial u}{\partial y}=\nu \frac{{\partial }^{2}u}{\partial {x}^{2}}-\lambda [{u}^{2}\frac{{\partial }^{2}u}{\partial {x}^{2}}+2uv\frac{{\partial }^{2}u}{\partial x\partial y}+{v}^{2}\frac{{\partial }^{2}u}{\partial {y}^{2}}]-\frac{\sigma {B}_{o}^{2}}{{\rho }_{f}}(\lambda v\frac{\partial u}{\partial y}+u),$$

3 $$u\frac{\partial T}{\partial x}+v\frac{\partial T}{\partial y}=\alpha \frac{{\partial }^{2}T}{\partial {y}^{2}}+\tau \{\frac{{D}_{T}}{{T}_{\infty }}{(\frac{\partial T}{\partial y})}^{2}+{D}_{B}(\frac{\partial C}{\partial y}\frac{\partial T}{\partial y})\},$$

4 $$u\frac{\partial C}{\partial x}+v\frac{\partial C}{\partial y}={D}_{B}(\frac{{\partial }^{2}C}{\partial {y}^{2}})+\frac{{D}_{T}}{{T}_{\infty }}(\frac{{\partial }^{2}T}{\partial {y}^{2}}).$$

where, *u* is the *x* component and *v* is the *y* component of the Maxwell fluid velocity. Also *g*, *D*_*T*_, *C*, *T*_∞_, *λ*, *ν*, *ρ*_*f*_, *C*_∞_, *T*, *σ*, *D*_*B*_ and *α* represents gravitational acceleration, thermophoretic diffusion coefficient, nanoparticle volume fraction, free stream temperature, relaxation time, the ratio between nanoparticle and original fluids heat capacities, kinematic viscosity, fluid density, free stream concentration, temperature, electrical conductivity, Brownian diffusion coefficient and thermal diffusivity, respectively. The subjected boundary conditions (BCs) are given by,

5 $${U}_{w}(x)=u={U}_{0}exp(\frac{x}{l}),\,{T}_{w}=T={T}_{\infty }+{T}_{0}exp(\frac{x}{2l}),\,\,v=0,$$

6 $${C}_{w}=C={C}_{\infty }+{C}_{0}\,{\exp }(\frac{x}{2l})\,{\rm{at}}\,y=0,$$

7 $$u\to 0,\,\,C\to {C}_{\infty },\,\,T\to {T}_{\infty },\,\,v\to 0\,\,{\rm{as}}\,\,y\to \infty .$$

The adequate transformations for the considered problem are taken as follows:

8 $$\begin{array}{rcl}\eta  & = & \sqrt{\frac{{U}_{0}}{2\nu l}}\,{\exp }(\frac{x}{2l})y,\,\,\psi =\sqrt{2l\nu {U}_{0}}\,f(\eta ){\exp }(\frac{x}{2l}),\,\,u{U}_{0}exp(\frac{x}{l})\,f^{\prime} (\eta ),\\ v & = & \sqrt{\frac{{U}_{0}\nu }{2l}}\,{\exp }(\frac{x}{2l})[\eta \,f^{\prime} (\eta )+f(\eta )],\,\theta (\eta )=\frac{T-{T}_{\infty }}{{T}_{0}\,{\exp }(\frac{x}{2l})},\,\,\varphi (\eta )=\frac{C-{C}_{\infty }}{{C}_{0}\,{\exp }(\frac{x}{2l})}.\end{array}$$

Substitution of (8) into (2)–(4) yields

9 $$\begin{array}{c}f{\prime}{\prime}{\prime} -2{f{\prime} }^{2}-2{M}^{2}f{\prime} +\beta (3ff{\prime} f^{\prime\prime} +\frac{\eta }{2}{(f{\prime} )}^{2}f^{\prime\prime} -2{(f{\prime} )}^{3}-\frac{1}{2}{f}^{2}f{\prime}{\prime}{\prime} )\\ \,\,\,\,\,\,\,+{M}^{2}\beta (\eta f{\prime} f^{\prime\prime} +ff^{\prime\prime} )+ff^{\prime\prime} =0,\end{array}$$

10 $$\theta ^{\prime\prime} +Pr(f\theta{\prime} +{N}_{t}{\theta{\prime} }^{2}-f{\prime} \theta +{N}_{b}\theta{\prime} \varphi{\prime} )=0,$$

11 $$\varphi ^{\prime\prime} +\frac{{N}_{t}}{{N}_{b}}\theta ^{\prime\prime} -PrLe(f{\prime} \varphi -f\varphi{\prime} )=0,$$

in which the incompressibility condition (1) is satisfied identically and the parameters *Le*, *M*, *N*_*b*_, *β*, *Pr*, *N*_*t*_ are the Lewis number, Hartman number, the Brownian motion parameter, Deborah number, Prandtl number and the thermophoresis parameter, respectively. The definitions of these numbers are,

12 $${M}^{2}=\frac{\rho {B}_{0}^{2}l}{\rho {U}_{w}},\,\beta =\frac{\lambda {U}_{w}}{l},\,{N}_{t}=\frac{\tau {D}_{T}{T}_{0}exp(x/2l)}{\nu {T}_{\infty }},\,Le=\frac{\alpha }{{D}_{B}},\,Pr=\frac{\nu }{\alpha }.$$

The non-dimensional BCs from Eqs (5)–(7) becomes

13 $$\begin{array}{c}f(0)=0,f{\prime} (0)=1,\varphi (0)=1,\,(0)=1,\\ f{\prime} (\infty )=0,\theta (\infty )=0,\varphi (\infty )=0.\end{array}\}$$

The heat and mass transfer rates in terms of local Sherwood, the local Nusselt numbers, and the local skin friction coefficient are defined by

14 $$Sh=\frac{x{j}_{i}}{{D}_{B}({C}_{\infty }-{C}_{w})},Nu=\frac{x{q}_{i}}{K({T}_{\infty }-{T}_{w})},{C}_{f}=\frac{2{\tau }_{i}}{\rho {U}_{w}^{2}},$$

where *j*_*i*_, *q*_*i*_, and *τ*_*i*_ are the mass, heat, and momentum fluxes from the surface. These are defined as follows:

15 $${j}_{i}=-\,{D}_{B}{(\frac{\partial C}{\partial y})}_{y=0},\,{q}_{i}=-\,K{(\frac{\partial T}{\partial y})}_{y=0},\,{\tau }_{i}=\mu {(\frac{\partial u}{\partial y})}_{y=0}$$

In dimensionless form they are represented as:

16 $$R{e}_{x}^{-\frac{1}{2}}\,Sh=-\sqrt{\frac{X}{2}}\varphi{\prime} (0),\,R{e}_{x}^{-\frac{1}{2}}N{u}_{x}=-\sqrt{\frac{X}{2}}\theta{\prime} (0),\,{(2Re)}^{\frac{1}{2}}\,{C}_{f}=\sqrt{2X}f^{\prime\prime} (0).$$

## Homotopy-Based Approach

The following methodology details should provide as a guide about OHAM aiming to solve nonlinear differential system (9)–(11) with BCs (13) and identify the variations of physical solutions of the differential system. In the framework of OHAM, we can choose auxiliary linear operators in the forms

17 $${ {\mathcal L} }_{1}[u(\eta :q)]=\frac{{d}^{3}u}{d{\eta }^{3}}-\frac{du}{d\eta },$$

18 $${ {\mathcal L} }_{2}[v(\eta :q)]=\frac{{d}^{2}u}{d{\eta }^{2}}-v.$$

Obviously, the operators satisfying the below assumptions

19 $${ {\mathcal L} }_{1}[{D}_{1}\exp (-\eta )+{D}_{2}exp(\eta )+{D}_{3}]=0,$$

20 $${ {\mathcal L} }_{2}[{D}_{4}\exp (-\eta )+{D}_{5}exp(\eta )]=0.$$

The corresponding auxiliary linear operator for *f*(*η*) is ${ {\mathcal L} }_{1}$ and ${ {\mathcal L} }_{2}$ corresponds to *θ*(*η*) and *ϕ*(*η*). In OHAM we also have flexibility to pick the initial solutions. It is mandatory that all initial solutions should satisfy the BCs (13). Therefore, we set the initial solutions as follows:

21 $${f}_{0}(\eta )=1-\exp (-{\lambda }_{a}\eta ),$$

22 $${\theta }_{0}(\eta )=\exp (-{\lambda }_{b}\eta ),$$

23 $${\varphi }_{0}(\eta )=\exp (-{\lambda }_{b}\eta ).$$

where *λ*_*a*_ = *λ*_*b*_ = 1. We have applied BVPh 2.0 to solve nonlinear differential system (9)–(11) with BCs (13). With linear operators (17) and (18) and initial solutions (21)–(23), the Eqs (9)–(11) with BCs (13) can be solved directly by using BVPh 2.0 in quite easy and convenient way.

## Results and Discussion

The OHAM approximations contains unknown convergence enhancing parameters ${c}_{0}^{f},\,{c}_{0}^{\theta }$ and ${c}_{0}^{\varphi }$. Liao^46^ proposed Minimum Error Method that can be used to compute values for convergence enhancing parameters. These optimal convergence control parameters ensure the fast convergence of OHAM solutions. The mechanism for the choice of optimal convergence enhancing parameters is explained by an illustrative example. Let us assume *β* = *N*_*b*_ = *M* = *N*_*t*_ = 0.1, *Le* = *Pr* = *λ* = 1.0, the optimal values of ${c}_{0}^{f},\,{c}_{0}^{\theta }$ and ${c}_{0}^{\varphi }\,$are computed through the minimization of squared residual error as shown in Table 1. It is noted that the total error is decreased by increasing the order of iteration. Optimal convergence-control parameters corresponding to 5th-order OHAM iteration are then used to check the convergence of our results at various orders of approximation. The OHAM iterations at various orders are shown in Table 2. The presented results demonstrate the high efficiency and reliability of OHAM series solutions. The graphical analysis has been accomplished for the flow pattern, concentration, temperature, the local Sherwood number and the local Nusselt number for various values of *β*, *Le*, *Pr*, *M*, *N*_*t*_ and *N*_*b*_.

**Table 1:** Choice of convergence enhancing parameters for *β* = *N*_*b*_ = *M* = *N*_*t*_ = 0.1, *Le* = *Pr* = 1.0.

*m*	${{\boldsymbol{c}}}_{{\bf{0}}}^{{\boldsymbol{f}}}$	${{\boldsymbol{c}}}_{{\bf{0}}}^{{\boldsymbol{\theta }}}$	${{\boldsymbol{c}}}_{{\bf{0}}}^{{\boldsymbol{\varphi }}}$	${{\boldsymbol{\varepsilon }}}_{{\boldsymbol{m}}}^{{\boldsymbol{t}}}$	*t* (seconds)
1	−1.02	−0.04	−1.48	0.15 × 10^−1^	0.968055
3	−1.19	−0.82	−1.48	0.37 × 10^−2^	11.357
5	−1.34	−0.91	−1.56	0.17 × 10^−2^	85.337

**Table 2:** Squared residual errors with ${c}_{0}^{f}=-\,1.34,\,{c}_{0}^{\theta }=-\,0.91,\,{c}_{0}^{\varphi }=-\,1.56$ for *β* = *N*_*b*_ = *M* = *N*_*t*_ = 0.1, *Le* = *Pr* = 1.0.

*m*	10	20	30
${\varepsilon }_{m}^{f}$	2.07 × 10^−7^	1.17 × 10^−7^	7.08 × 10^−8^
${\varepsilon }_{m}^{\theta }$	0.28 × 10^−4^	8.68 × 10^−6^	4.77 × 10^−6^
${\varepsilon }_{m}^{\varphi }$	0.62 × 10^−3^	0.22 × 10^−3^	0.13 × 10^−3^
${\varepsilon }_{m}^{t}$	0.64 × 10^−3^	0.23 × 10^−3^	2 × 10^−3^
*t* (seconds)	59.2263511	707.5565964	5623.3201208

**Figure 2:**
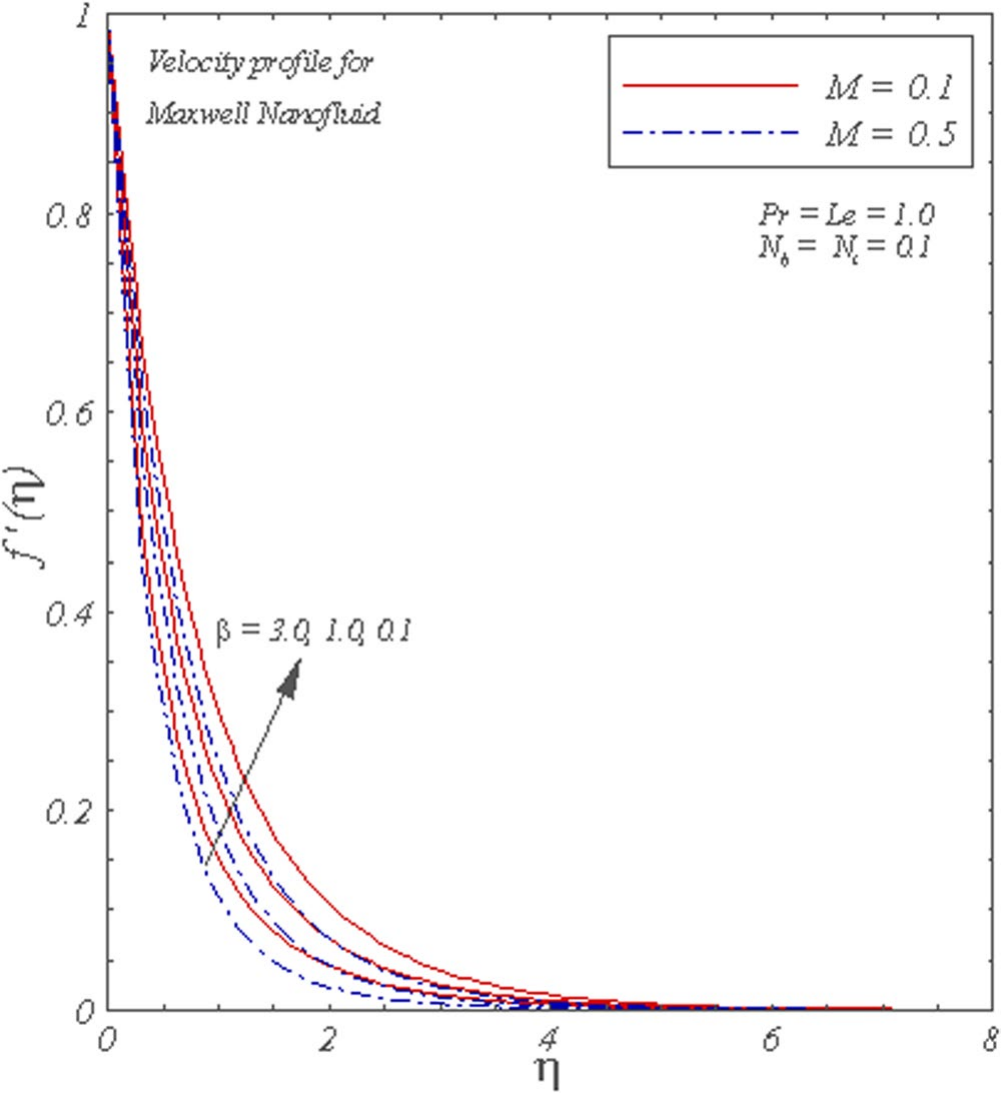
Graphs of *f* ′(*η*) for different *β* and *M*.

Figure 2 illustrates the impact of *β* on the velocity graph for different *M*. It is visible that fluid flow is maximum in the ambient fluid for smaller *β*. However, the fluid changes its properties from Newtonian to non-Newtonian characteristics for higher *β*, and hence the flow shows decreasing behavior. The boundary layer thickness reduces as we increase *β* and *M*.

**Figure 3:**
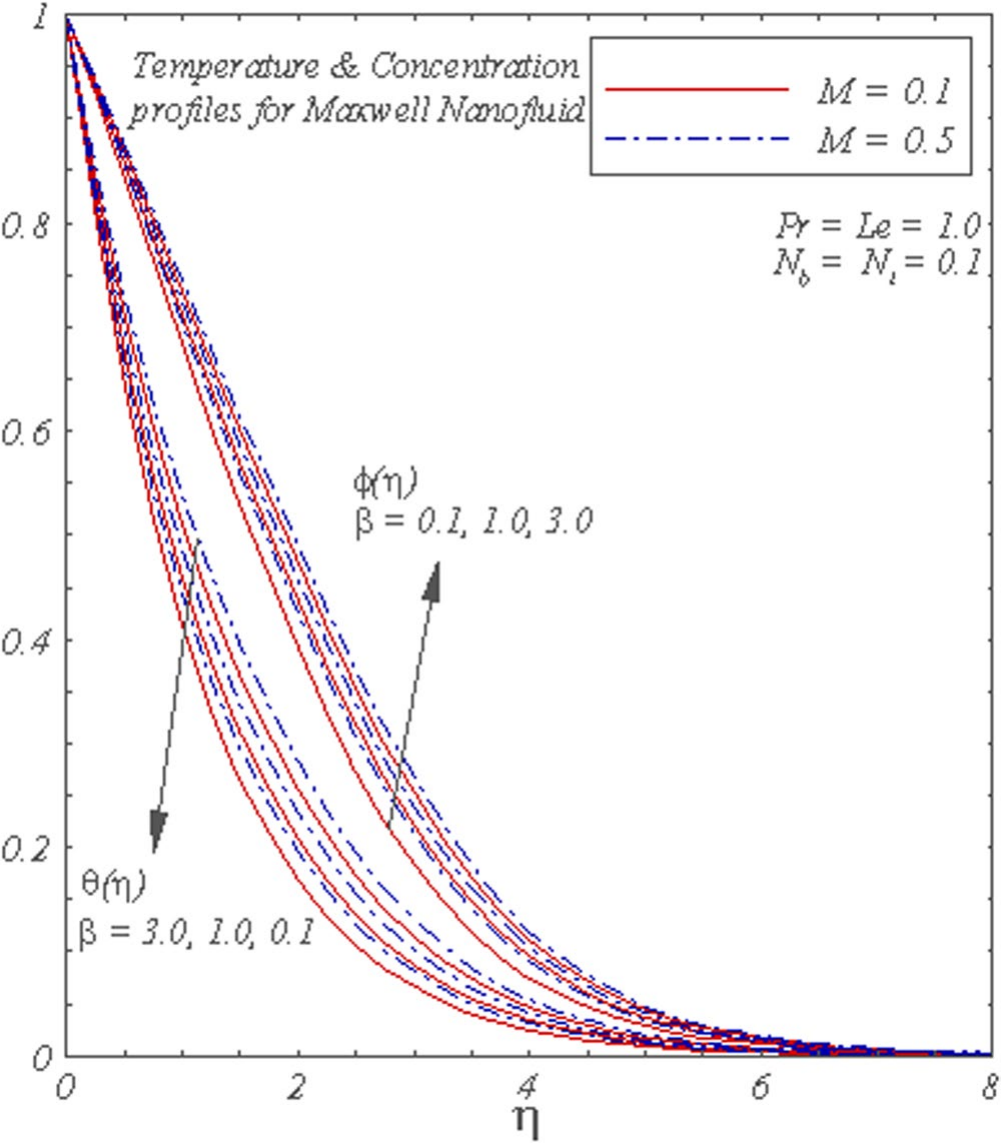
Graphs of *θ*(*η*) and *ϕ*(*η*) for different values of *β* and *M*.

Figure 3 displays the influence of *β* and *M* on concentration and temperature graphs. It is observed that the increase in *β* and *M* enhances concentration and temperature.

**Figure 4:**
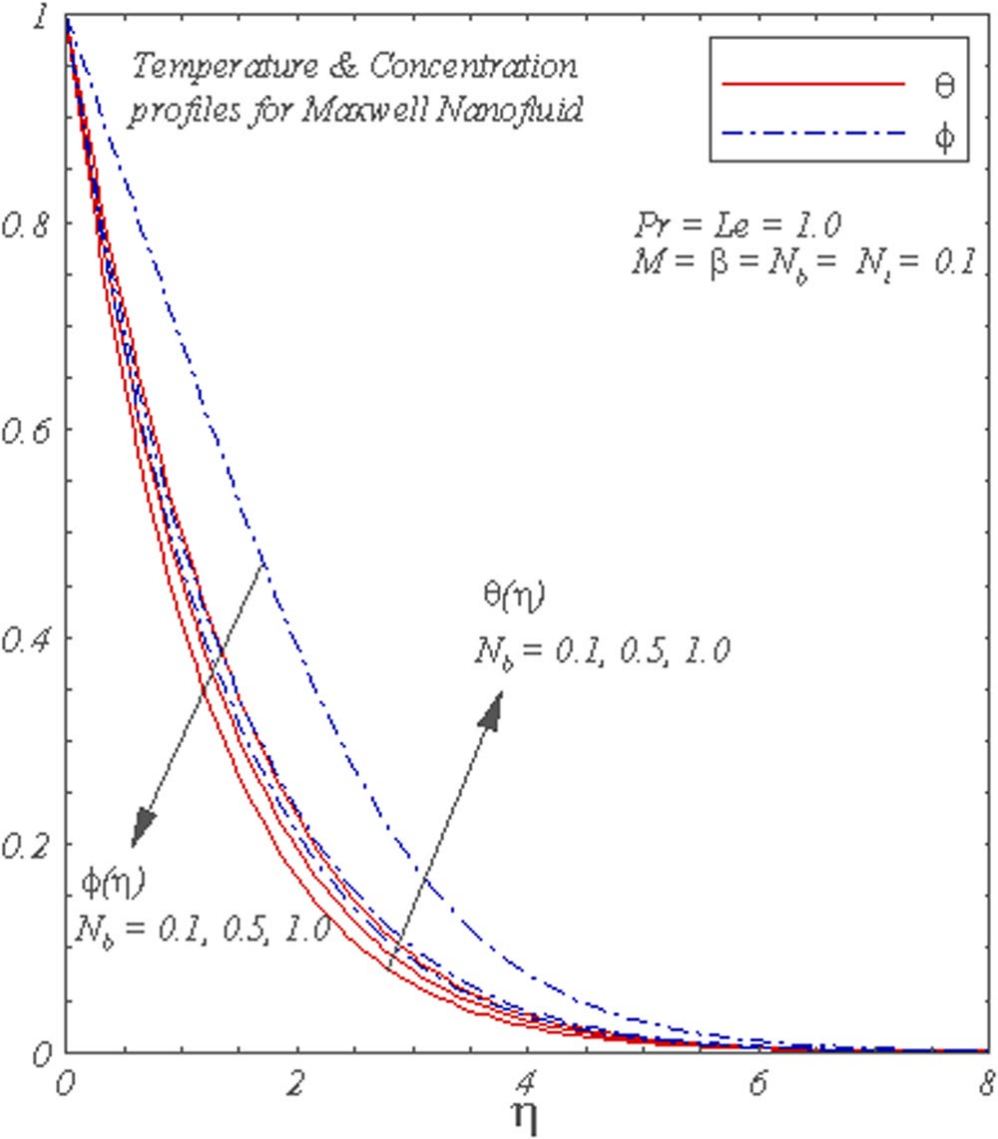
Graphs of *θ*(*η*) and *ϕ*(*η*) for different *N*_*b*_.

Figure 4 describes the dimensionless concentration and temperature for various values of *N*_*b*_. The temperature increases with the increase of *N*_*b*_ but concentration decreases in the boundary layer region.

**Figure 5:**
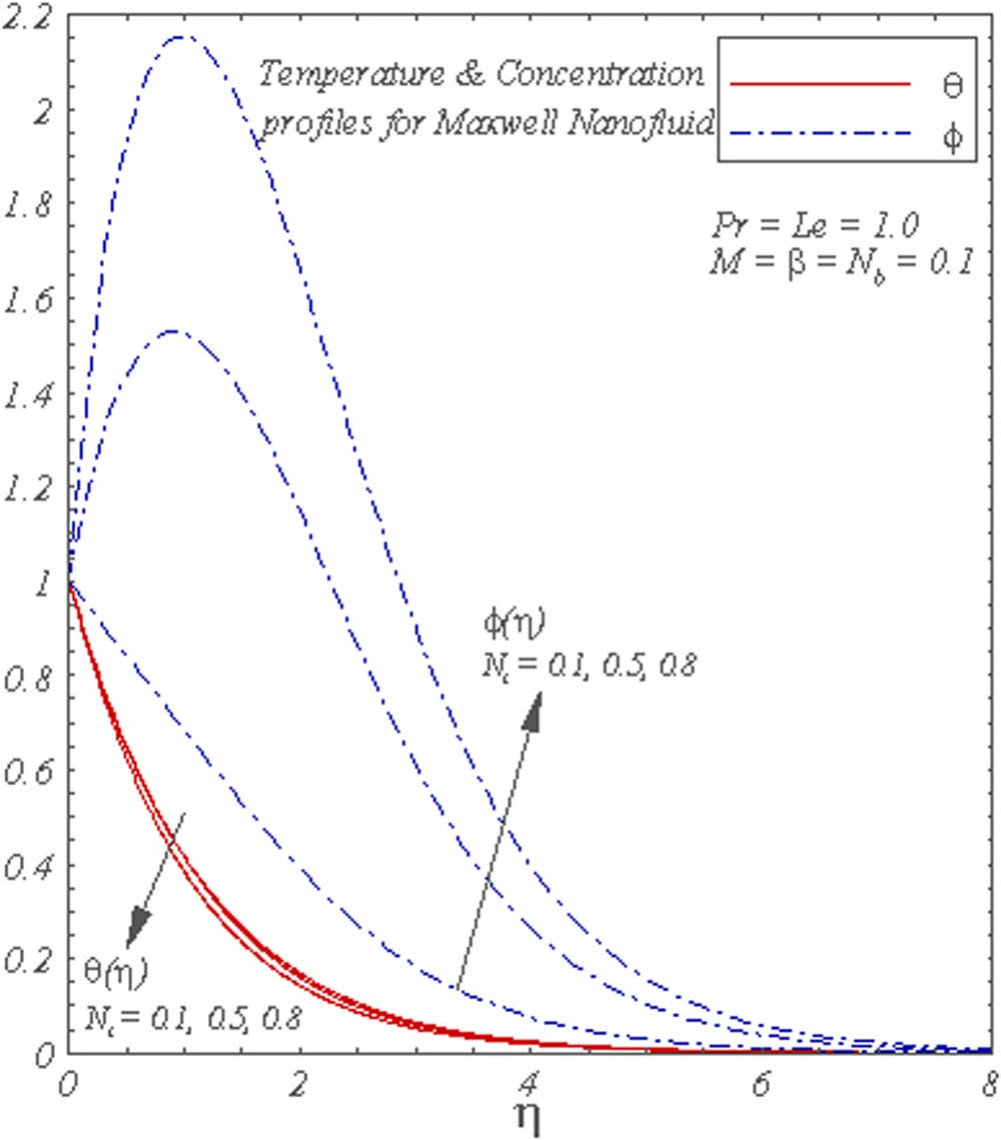
Graphs of *θ*(*η*) and *ϕ*(*η*) for different *N*_*t*_.

Figure 5 illustrates that due to increase in the values of *N*_*t*_, the concentration profile increases but temperature decreases. The overshoot in the concentration is observed that is highest concentration occurs in the ambient fluid but not at the surface.

**Figure 6:**
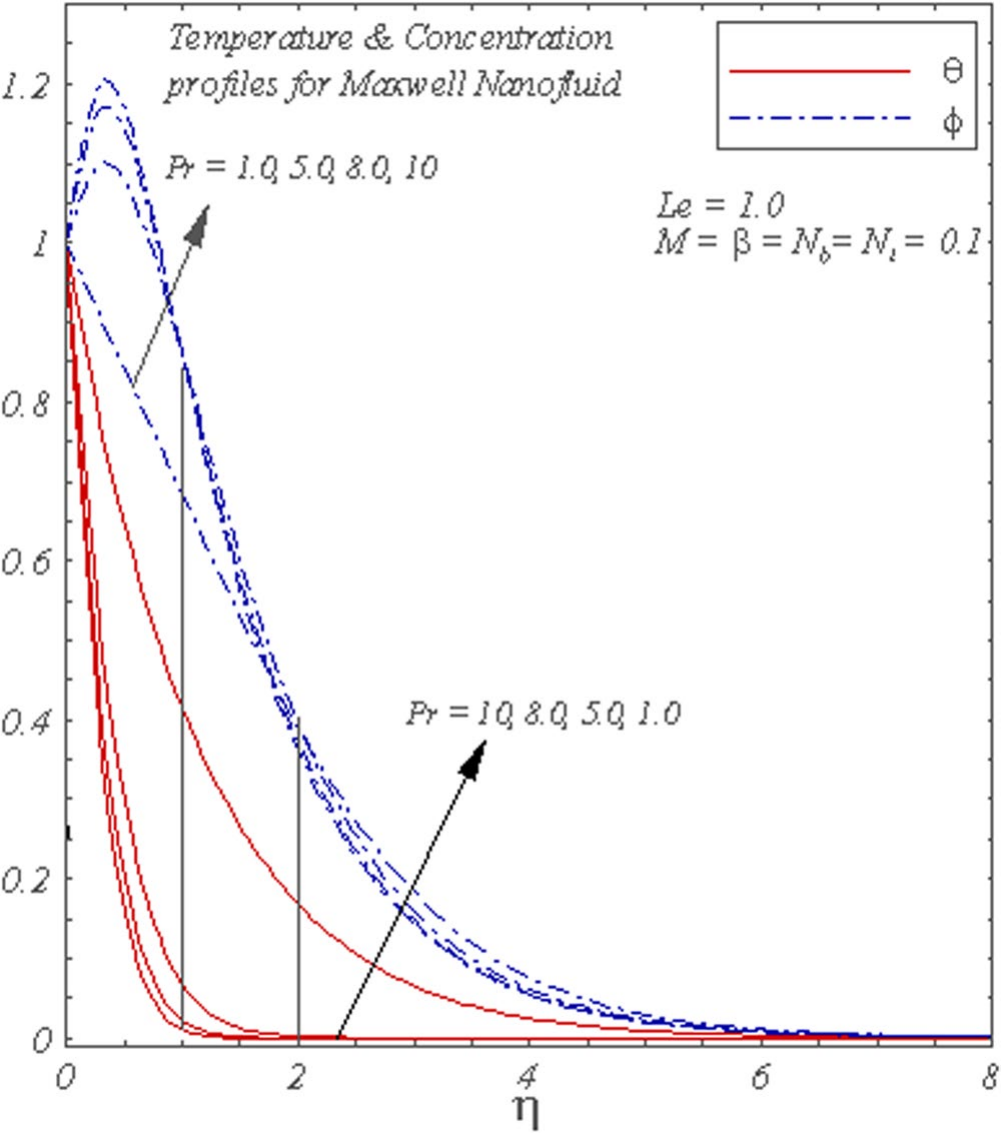
Graphs of *θ*(*η*) and *ϕ*(*η*) for different *Pr*.

Figure 6 describes the influence of *Pr* on the concentration and temperature profiles. Temperature is a decreasing function of *Pr*. The dimensionless temperature decreases for higher *Pr* and hence the thermal boundary layer reduces. Concentration overshoot is observed near the wall.

**Figure 7:**
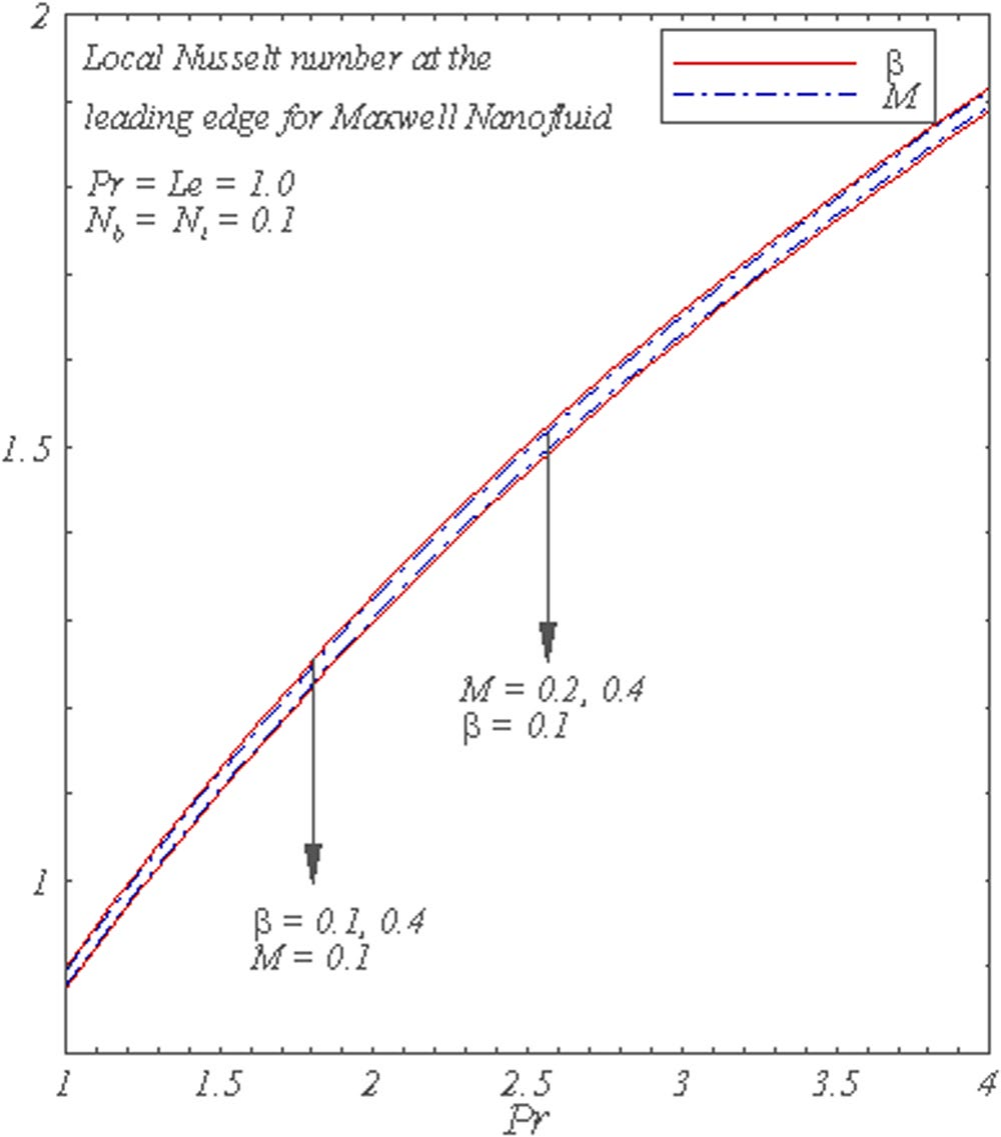
Graphs of the local Nusselt number for *Pr* with varying *M* and *β*.

Figure 7 displays the local Nusselt number for several values of *β* and *M*. The local Nusselt number is an increasing function of *Pr* but the increase in *β* and *M* decreases the local Nusselt number.

**Figure 8:**
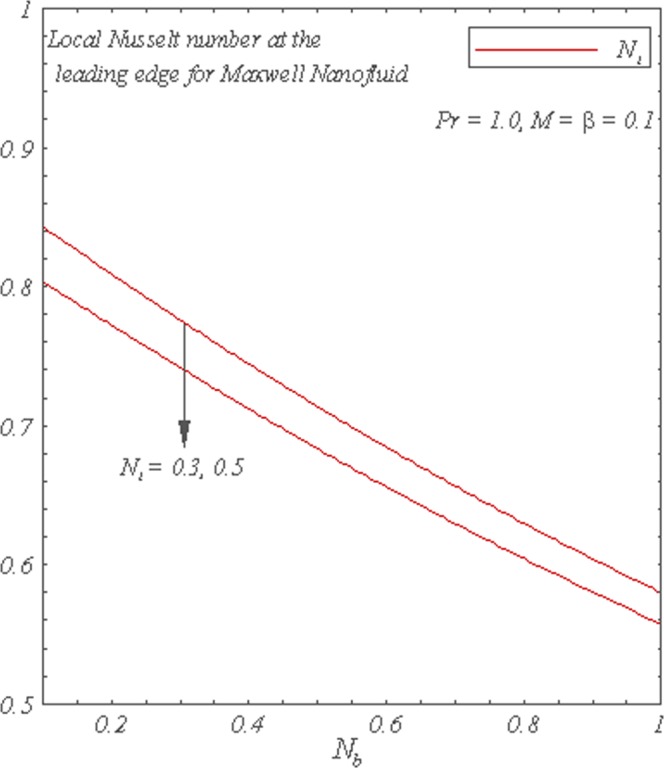
Graphs of the local Nusselt Number for *N*_*b*_ with varying *N*_*t*_.

**Figure 9:**
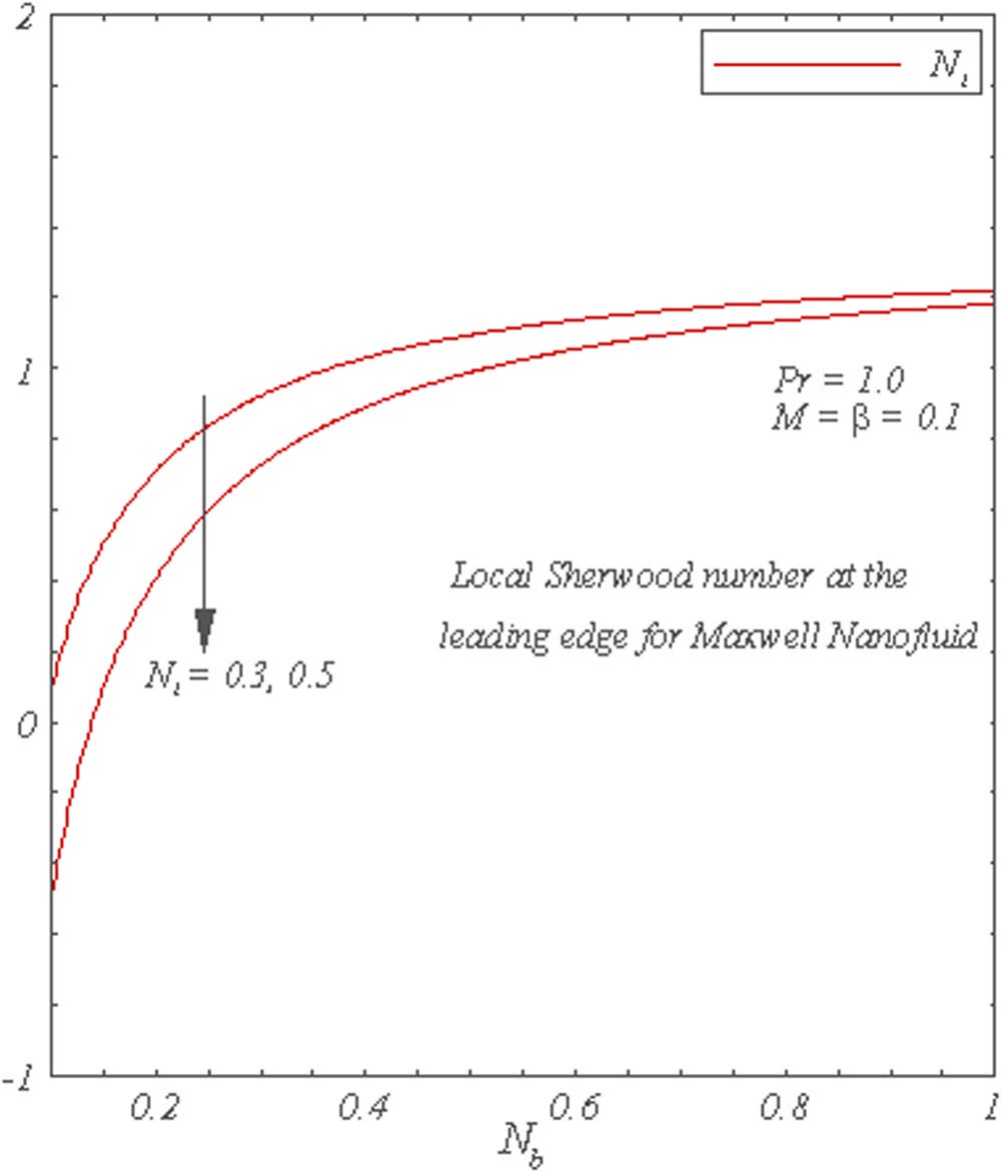
Graphs of the local Sherwood Number for *N*_*b*_ with varying *N*_*t*_.

Figure 8 shows that the local Nusselt number is a decreasing function of *N*_*b*_. The increase in either *N*_*t*_ or *Le* decreases the local Nusselt number. The local Sherwood number is plotted as a function of *N*_*b*_ in Fig. 9. It can be seen that local Sherwood number is an increasing function of *N*_*b*_ and *Le*. However, the increase in *N*_*t*_ decreases the local Sherwood number.

**Figure 10:**
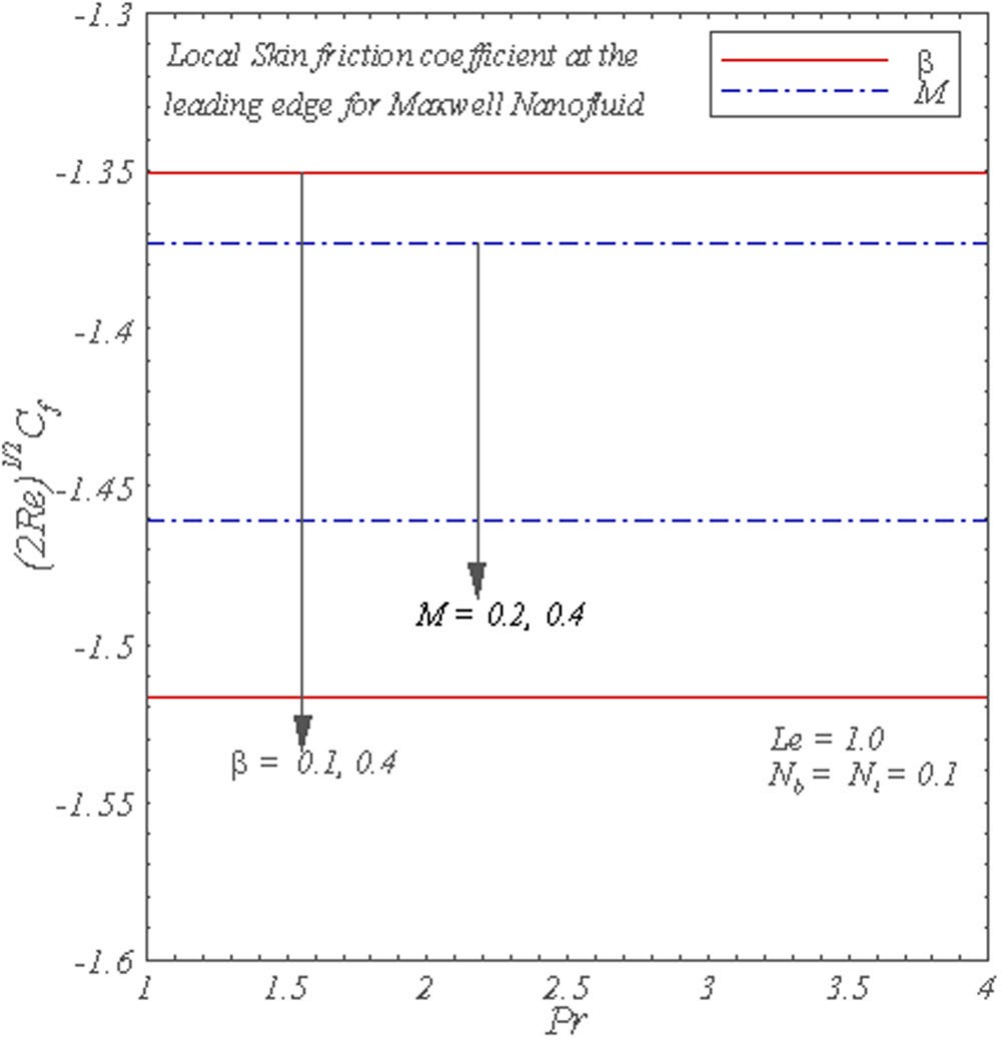
Graphs of the local skin friction coefficient with varying *M* and *β*.

Figure 10 exhibits the local skin friction coefficient as a function of Prandtl number. The change in Prandtl number does not affect the local skin friction coefficient. It is also observed the increase in *β* and *M* reduces the local skin friction coefficient.

## Conclusions

The main points of presented analysis are given below.

- Increase in *β* and *M* enhances the flow.
- Temperature is enhanced through increase in *β*, *M* and *N*_*b*_ whereas it decreases due to increase in *N*_*t*_ and *Pr*.
- The increase in the values of *β*, *M* and *N*_*t*_ leads to an increase in the volumetric concentration profile, whereas quite opposite is true for *N*_*b*_.
- The local Nusselt number increases with an increase in *Pr*. However, local Nusselt number decreases with the increase in *M*, *β*, *L*_*e*_ and *N*_*t*_.
- The local Sherwood number is increasing function of *N*_*b*_ and *L*_*e*_, whereas it decreases for an increase in *N*_*t*_.
